# The Role of the Extracellular Matrix in Cancer Stemness

**DOI:** 10.3389/fcell.2019.00086

**Published:** 2019-07-05

**Authors:** Sameera Nallanthighal, James Patrick Heiserman, Dong-Joo Cheon

**Affiliations:** Department of Regenerative and Cancer Cell Biology, Albany Medical College, Albany, NY, United States

**Keywords:** extracellular matrix, cancer stem cells, self-renewal, chemoresistance, integrin

## Abstract

As our understanding of cancer cell biology progresses, it has become clear that tumors are a heterogenous mixture of different cell populations, some of which contain so called “cancer stem cells” (CSCs). Hallmarks of CSCs include self-renewing capability, tumor-initiating capacity and chemoresistance. The extracellular matrix (ECM), a major structural component of the tumor microenvironment, is a highly dynamic structure and increasing evidence suggests that ECM proteins establish a physical and biochemical niche for CSCs. In cancer, abnormal ECM dynamics occur due to disrupted balance between ECM synthesis and secretion and altered expression of matrix-remodeling enzymes. Tumor-derived ECM is biochemically distinct in its composition and is stiffer compared to normal ECM. In this review, we will provide a brief overview of how different components of the ECM modulate CSC properties then discuss how physical, mechanical, and biochemical cues from the ECM drive cancer stemness. Given the fact that current CSC targeting therapies face many challenges, a better understanding of CSC-ECM interactions will be crucial to identify more effective therapeutic strategies to eliminate CSCs.

## Introduction: ECM as a CSC Niche

The extracellular matrix (ECM) is a major structural component of the tumor microenvironment and comprised of a network of biochemically distinct components, including fibrous proteins, glycoproteins, proteoglycans, and polysaccharides. The ECM is a highly dynamic structure, constantly undergoing a remodeling process where ECM components are deposited, degraded, or modified (Lu et al., 2012). Increasing evidence suggests that the ECM serves as a niche for normal and cancer stem cells (CSCs). CSCs, also called tumor-initiating cells, are a small population of cells within tumors that have capabilities of self-renewal properties, tumor initiation and chemoresistance (Kreso and Dick, 2014; Batlle and Clevers, 2017). As one of the CSC niches, the ECM provides both structural and biochemical support to regulate proliferation, self-renewal, and differentiation of CSCs. In this review, we will cover the current understanding of how different ECM components affect the cancer “stemness” phenotype.

## Categories of ECM Proteins and Their Role in Cancer Stemness

### Fibrous ECM Proteins

**FIGURE 1:**
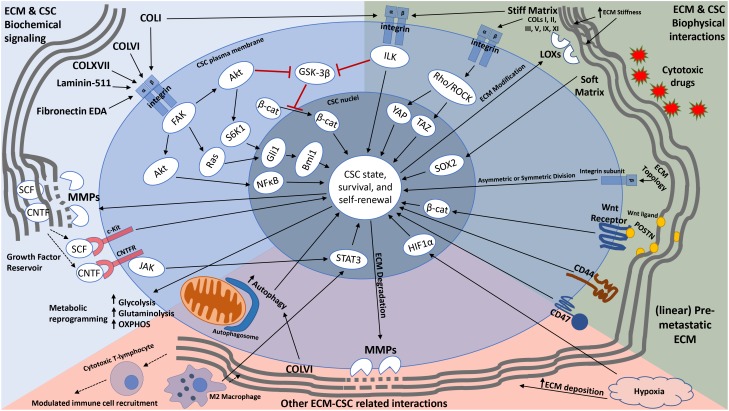
Schematic representation of how the ECM modulates cancer stemness. In addition to providing cues that transform non-CSCs into CSCs (through EMT) and maintain a stemness state, the ECM can modulate CSC metabolism, influence immune cell recruitment, and serve as a reservoir for growth factors and other signaling molecules that aid in CSC self-renewal and maintenance. Furthermore, the ECM provides not only a physical barrier to CSCs from cytotoxic drugs, but also anchorage sites for CSCs for cell division and metastatic colonization. CSCs are also able to modify their local ECM through upregulation of ECM degrading and modifying enzymes (such as MMPs and LOXs). Solid long arrows represent downstream signaling activation or event, solid short arrows represent elevated activity or expression, dotted arrows represent growth factor release or immune cell migration, red lines with flat heads represent inhibition.

Collagens constitute the main structural element of the ECM and are the most copious type of fibrous proteins within the interstitial ECM. Collagens play a role in tissue development by providing mechanical strength, altering cell adhesion, promoting cell migration (Frantz et al., 2010). Studies have reported that several collagens (e.g., COL3A1, COL4A2, COL7A1, COL17A1) are overexpressed by CSCs (Table 1). Multiple collagen subtypes have been shown to increase epithelial-mesenchymal transition (EMT), tumor-initiating potential, drug resistance and self-renewal of CSCs (Table 1 and Figure 1).

**Table 1:** The role of different ECM proteins in cancer stemness.

			Role in cancer stemness	References
**Fibrous proteins**	Collagen	Type I collagen	Maintains the self-renewal of mouse ES cells through Bmi-1 via α2β1 integrin and DDR1; promotes EMT; CD133^+^ glioblastoma CSCs are localized to type I collagen-rich perivascular niche; GBM cells cultured on type I collagen maintain stemness and tumorigenicity; increases expression of CD133 and Bmi1, EMT and clonogenicity in colorectal CSCs through α2β1 integrin; enhances tumor-initiating potential and self-renewal of ALDH^+^ pancreatic CSCs through β1integrin and FAK signaling.	Kirkland, 2009; Medici and Nawshad, 2010; Suh and Han, 2011; Motegi et al., 2014; Begum et al., 2017
	
		Type III collagen	COL3A1 is highly expressed in ALDH1A1^+^ topotecan-resistant ovarian CSCs.	Januchowski et al., 2016
	
		Type IV collagen	COL4A2 is highly expressed in CD133^+^/CD44^+^ prostate cancer spheroids; Head and neck CSCs grown on type IV collagen-coated plates grow much faster than in suspension and maintain CSC traits.	Lim et al., 2012; Oktem et al., 2014a
	
		Type VII collagen	COL7A1 is highly expressed in CD133^+^/CD44^+^ prostate cancer spheroids.	Oktem et al., 2014b
	
		Type XI collagen	COL11A1 promotes chemoresistance in ovarian cancer; COL11A1 increases the expression of TWIST1, a master EMT regulator directly involved in generating a breast CSC phenotype.	Vesuna et al., 2009; Wu et al., 2015, 2017; Rada et al., 2018
	
		Type XVII collagen	COL17A1 is upregulated in lung cancer spheroids and required for the maintenance of CSC characteristics and EMT phenotypes; works with laminin 332 to maintain CSC characteristics and EMT phenotype in lung cancer.	Liu et al., 2016, 2018

**Glycoproteins**	Fibulin	Fibulin-1	Fibulin-1 promotes doxorubicin resistance in breast cancer cells.	Pupa et al., 2007
	
		Fibulin-3	Fibulin-3 inhibits self-renewal of ALDH^+^ lung CSCs and EMT through IGF1R signaling; suppresses self-renewal of pancreatic CSCs by downregulating c-MET and ALDH1 expression; works as a downstream effector of HIF2α to stimulate breast CSC self-renewal.	Kim et al., 2014a,b; Kwak et al., 2016
	
	Fibrillin	Fibrillin-1	Fibrillin-1 supports growth, self-renewal, attachment and maintenance of human ES cells; increases the number and clonogenic potential of MSCs; promotes the expansion of HSCs.	Soteriou et al., 2013; Smaldone et al., 2016a,b
	
	Laminin	Laminin 511	Laminin 511 supports self-renewal of mouse ES cells and breast CSCs through the interaction with integrin α6β1.	Domogatskaya et al., 2008; Chang et al., 2015
		Laminin 332 (laminin 5)	Laminin 332 maintains CSC characteristics and EMT phenotype in lung cancer; supports stemness of human hepatic CSCs by promoting quiescence, chemoresistance, the number of side population, and *in vivo* tumor growth in a mTORC2-dependent manner.	Govaere et al., 2016; Liu et al., 2016
	
		Laminin alpha 2	Laminin α2 chain is expressed in the perivascular niche and crucial for survival, proliferation, and self-renewal of glioblastoma stem cells.	Lathia et al., 2012
	
		Laminin alpha 5	Laminin α5 is produced by human pluripotent stem cells (hPSC) and crucial for hPSC self-renewal.	Laperle et al., 2015
	
	Fibronectin	FN	FN is a marker for EMT-driven cancer stemness and induces EMT; increases the adhesion, proliferation and chemoresistance of glioma stem cells as well as their capacity for differentiation through the integrin/FAK/paxillin/AKT signaling pathway.	Li et al., 2017; Yu et al., 2018
	
		EDA-FN	EDA-FN is required for the sphere formation capacity, clonogenicity, and tumorigenic capacity of CD133^+^/CD44^+^ colon CSCs; CD133^+^/CD44^+^ colon CSCs express higher levels of the EDA receptor integrin α9β1 than CD133^−^/CD44^−^ non-CSCs and EDA binding to integrin α9β1 activates FAK/ERK/β-catenin signaling pathway to maintain stemness.	Ou et al., 2013
	
		EDB-FN	EDB-FN is crucial for mammosphere-forming ability, expression of CSC markers, self-renewal genes, drug resistance genes, and EMT markers, and *in vivo* tumorigenicity of breast CSCs.	Sun et al., 2015
	
	Vitronectin		Vitronectin supports sustained self-renewal and pluripotency of human ES cells in defined media; downregulates self-renewal genes and induces differentiation of prostate CSCs in an αVβ3 integrin–dependent manner.	Braam et al., 2008; Hurt et al., 2010
	
	Fibrinogen		Soft 3D fibrin gels promote formation of tumor spheroids and tumorigenic potential of melanoma CSCs.	Liu et al., 2012
	
	Tenascin	Tenascin-C	Oct4^+^/TNC^+^ neuroblastoma CSCs, found in the perivascular niche, display a high degree of plasticity and serve as progenitors of tumor-derived endothelial cells; TNC is co-expressed with CD133, a marker for GBM CSCs, in primary GBM tissues; TNC^+^ GBM CSCs exhibit the strongest sphere forming capacity regardless of CD133 status; promotes growth of GBM CSCs through α2β1 integrin-mediated upregulation of NOTCH ligand Jagged1 and other NOTCH signaling components; strongly enhances the expression of LGR5 and MSI1, the WNT and NOTCH signaling components that provide essential signals to stem cells, thereby promoting the survival and outgrowth of pulmonary micrometastases; increases side population, sphere formation, and chemoresistance of melanoma CSCs.	Fukunaga-Kalabis et al., 2010; Oskarsson et al., 2011; Pezzolo et al., 2011; Nie et al., 2015; Sarkar et al., 2017
	
	Secreted Protein Acidic and Rich in Cysteine (SPARC)		Overexpressed in endometrial CSCs; Most abundantly secreted by non-prostate CSCs and enhances the invasiveness and metastatic dissemination of prostate CSCs in a paracrine manner; plays a key role in maintaining dormancy of prostate cancer cells by upregulating BMP7 in bone marrow stromal cells; SPARC is highly expressed by HSCs that recently colonized the bone marrow. HSCs in a SPARC-deficient niche show an accelerated return to quiescence, thereby becoming resistant to serial 5-FU treatment.	Ehninger et al., 2014; Mateo et al., 2014; Yusuf et al., 2014; Sharma et al., 2016
	
	Periostin (POSTN)		POSTN promotes a stem cell-like trait and a mesenchymal phenotype in human mammary epithelial cells and breast cancer cells; plays an essential role in the crosstalk between CSCs and their niche to permit metastatic colonization; recruits Wnt ligands and increases Wnt signaling in breast CSCs, thereby promoting CSC maintenance and expansion; POSTN and its receptor αVβ3 integrin are highly expressed in CSC-enriched basal-like breast cancer; POSTN–β3 integrin signaling is required for the maintenance of breast CSCs by activating the ERK signaling pathway and regulating NF-kB–mediated transcription of IL6 and IL8; Glioma stem cells secrete POSTN to recruit M2 tumor-associated macrophages through αVβ3 integrin to support tumor growth; Secreted POSTN promotes GBM stem cell invasion and engraftment through αVβ3 and αVβ5 integrins.	Malanchi et al., 2011; Wang et al., 2013; Mikheev et al., 2015; Zhou W.C. et al., 2015; Lambert et al., 2016
	
	Thrombospondin	Thrombospondin 1 (TSP1)	TSP1 inhibits stem cell self-renewal by downregulating the expression of self-renewal genes through its receptor CD47 in primary murine endothelial cells; decreases the expression of self-renewal genes and sphere-forming capacity in human colon cancer (HCT116), non-small cell lung cancer (A549), and cervical cancer (HeLa) cell lines; CD47, a TSP1 receptor, is highly expressed in circulating hematopoietic stem cells, leukemia cells, breast CSCs, pancreatic CSCs, and AML leukemia stem cells and required for self-renewal of these CSCs.	Jaiswal et al., 2009; Majeti et al., 2009; Kaur et al., 2013; Cioffi et al., 2015; Zhang et al., 2015a; Zheng et al., 2015; Kaur et al., 2016
	
	Mucin	Mucin 1	MUC1 is highly expressed in AML stem cells, pancreatic CSCs, and breast CSCs; MUC1 overexpression increases stem cell properties in cord blood CD34^+^ cells and breast cancer cells; MUC1 is overexpressed and hypoglycosylated in the side population of MCF7 breast cancer cells; Staurosporine-induced apoptosis activates CD44^+^/CD24^−^ breast CSCs by upregulating MUC1 and EpCAM.	Engelmann et al., 2008; Fatrai et al., 2008; Curry et al., 2013; Stroopinsky et al., 2013; Zhou N. et al., 2015
	
		Mucin 4	MUC4 stabilizes HER2 expression and maintains ovarian CSCs; increases CD133^+^ pancreatic CSCs and confers gemcitabine resistance.	Mimeault et al., 2010; Ponnusamy et al., 2011
	
		Mucin 16 (CA125)	High levels of MUC16 are associated with poor clinical outcome and CSC-like properties; C-terminal domain of MUC16 enriches pancreatic CSCs through JAK2-mediated upregulation of LMO2 and NANOG.	Das et al., 2015; Zhang et al., 2015b
	
	Nidogen (entactin)	NID1	Nidogen-1 promotes EMT and cisplatin resistance in ovarian cancer cells	Zhou et al., 2017

**Proteoglycans**	Syndecan (CD138)	Syndecan-1	Loss of syndecan-1 in epithelial cells induces a mesenchymal phenotype; Shedding of syndecan-1 by MMP7 promotes chemoresistance; Syndecan-1 induces CSC phenotype via NF-kB/IL-6/STAT3 and Wnt signaling pathways.	Kato et al., 1995; Ibrahim et al., 2013; Wang et al., 2014
	
	Glypican	Glypican-3	Glypican-3 promotes self-renewal of hepatocellular CSCs.	Sun et al., 2017
	
		Glypican-4	Knockdown of GPC4 sensitizes pancreatic cancer cells to 5-FU and inhibits stem cell–like properties by suppressing Wnt/β-catenin pathway.	Cao et al., 2018
	
	Small leucin-rich proteoglycans (SLRP)	Decorin	Suppresses tumor cell growth, migration, angiogenesis, and metastasis in melanoma, osteosarcoma, and breast cancer; inhibits neural stem cell differentiation; inhibits ES cell self-renewal but promotes trophoblast stem cell self-renewal and commitment; suppresses the numbers of hematopoietic stem cells in the bone marrow and spleen; glioblastoma and neuroblastoma CSCs produce high levels of decorin to acquire temozolomide resistance and a quiescent phenotype.	Grant et al., 2002; Reed et al., 2005; Barkho et al., 2006; Shintani et al., 2008; Stock et al., 2011; Ichii et al., 2012; Farace et al., 2015; Nandi et al., 2018
	
		Lumican	Glioblastoma and neuroblastoma CSCs produce high levels of lumican and decorin to acquire temozolomide resistance and a quiescent phenotype.	Farace et al., 2015
	
		Biglycan	Biglycan is highly expressed in colon CSCs and promotes chemoresistance of colon cancer cells by activating NF-kB signaling.	Fang et al., 2010; Liu et al., 2018
	
		Asporin	Asporin inhibits TGF-β1-induced EMT and expansion of breast CSCs.	Maris et al., 2015
	
	Versican		High levels of versican are detected in CD133^+^/CD44^+^ prostate CSC spheroids; The C-terminal G3 domain of versican enhances self-renewal of breast CSCs and confer chemoresistance through EGFR/AKT/GSK-3β signaling.	Du et al., 2013; Oktem et al., 2014b
	
	Aggrecan		Aggrecan is expressed by neural stem cells and its expression is decreased upon differentiation; CD133^+^/CD44^+^ prostate CSC spheroids express high levels of aggrecan.	Kabos et al., 2004; Oktem et al., 2014a
	
	Testican		Testican-1 mediates EMT and confers acquired resistance to lapatinib in HER2-positive gastric cancer	Kim et al., 2014c

**Non-proteoglycan polysaccharides**	Hyaluronan (HA)		Breast CSCs produce high levels of HA; HA promotes the interaction of breast CSCs with tumor-associated macrophages to activate other stromal cells that augment the growth of CSCs; Excessive HA production promotes acquisition of CSC properties via Twist and the TGF-β-Snail signaling axis in breast cancer; HA-CD44 interaction induces Nanog-Stat-3 interaction, resulting in multidrug resistance in breast and ovarian cancer; HA–CD44 interaction stimulates stem cell marker expression, stemness properties and chemoresistance in head and neck CSCs.	Bourguignon et al., 2008, 2012; Okuda et al., 2012; Chanmee et al., 2014; Shiina and Bourguignon, 2015

### Glycoproteins

Glycoproteins, which make the ECM a cohesive network of molecules by linking cells together with structural components, include fibulin, fibrillin, laminin, fibronectin, vitronectin, tenascin-C, Secreted Protein Acidic and Rich in Cysteine (SPARC), periostin (POSTN), thrombospondin, mucins (MUCs) and nidogen (Table 1). CSCs overexpress several glycoproteins (e.g., tenascin-C, POSTN, MUC1) and their receptors (e.g., integrins αVβ3 and α9β1, CD47). Adhesive glycoproteins bind to integrins, non-integrin receptors, growth factors, and other ECM components to activate downstream signaling pathways to regulate EMT, self-renewal, and drug resistance of CSCs (Table 1). For example, fibronectin, a major adhesive ECM glycoprotein that attaches cells to a variety of ECM components, has been shown to increase EMT, self-renewal, expression of CSC markers and drug resistance of CSCs. Laminins, another class of adhesive glycoproteins that constitute structural scaffolding of all basement membranes, support self-renewal of CSCs through their interaction with integrins. Some glycoproteins have dual roles in cancer stemness depending on the cancer type. For instance, fibulin-3, an ECM glycoprotein associated with basement membranes, inhibits self-renewal in lung and pancreatic CSCs while stimulating breast CSC self-renewal (Table 1).

### Proteoglycans

Proteoglycans are glycosylated proteins composed of a core protein and one or several covalently attached sulfated glycosaminoglycan chains and are present in the ECM of connective tissues. Proteoglycans play a crucial role in ECM assembly and cell signaling. They bind to growth factors, cytokines and other ECM molecules and act as co-receptors to assist ligand and cell surface binding to modulate downstream signaling. Several proteoglycans (e.g., decorin, lumican, biglycan, versican, aggrecan) are highly expressed by CSCs and their roles in cancer stemness are summarized in Table 1.

### Polysaccharides

Polysaccharides, a chain of monosaccharide repeats linked through glycosidic bonds, fill the interstitial space and buffer physical stress on the ECM. Hyaluronic acid (HA or “hyaluronan”) is a high-molecular-mass polysaccharide that constitutes a major component of interstitial gels, especially in soft connective tissues. In tumors, HA is produced by both tumor stroma and tumor cells, and its binding to the cellular receptor CD44 activates intracellular signaling (e.g., PI3K/Akt and Erk pathways, RhoA and Rac, Ras, NF-kB and Src signaling) to promote cell survival, cancer stemness, motility and invasion by cytoskeletal reorganization. High levels of HA are produced by CSCs and HA–CD44 interaction has been shown to promote acquisition of CSC characteristics and chemoresistance in breast, ovarian and head and neck CSCs (Table 1).

## ECM Provides Physical and Mechanical Cues to Drive Cancer Stemness

### Physical Properties

Physical properties of the ECM such as rigidity, porosity and topography impact various anchorage-dependent CSC functions. The interstitial ECM, mainly composed of collagens, proteoglycans and hyaluronan, provides a physical barrier that hinders the transport of solutes, water and chemotherapeutic drugs. In this regard, it has been shown that cisplatin, a chemotherapeutic drug frequently used to treat various solid tumors, extensively binds to collagen fibers in tumors (Chang et al., 2016). Binding of chemotherapeutic drugs to the ECM prevents drug penetration into tumors, thereby increasing CSC survival. The ECM also provides sites for adhesion of CSCs in the tumor microenvironment. ECM-CSC interaction via CSC receptors such as integrins (e.g., β1, α6, β3, β4), discoidin domain receptors (DDR1, DDR2), CD44 (HA receptor) and CD47 (thrombospondin 1 receptor) enhances CSC properties. For example, CSCs bind to HA through CD44 and this increases not only the expression of stemness factors NANOG and SOX2 but also MDR1 (Multi Drug Resistance 1) expression and drug resistance in breast and ovarian CSCs (Bourguignon et al., 2008). The ECM also provides anchorage and homing sites for CSCs in pre-metastatic niches, initiating metastatic colonization and organotropism of cancer cells. For instance, infiltrating breast tumor cells induce the expression of POSTN in the stroma of the secondary target organ (e.g., lung). By recruiting Wnt ligands and increasing Wnt signaling in CSCs, POSTN sustains CSC population in the secondary site and promotes metastatic colonization (Malanchi et al., 2011). Changes in the ECM topology also affects CSC self-renewal by controlling the balance between symmetric and asymmetric cell divisions. The spatial distribution of the ECM has been shown to guide the orientation of the cell division axis by controlling the location of actin polymerization at the membrane through focal adhesions and the segregation of cortical components in the interphase (Thery et al., 2005). The β1 sub-family of integrins also regulates stem cell self-renewal by controlling the balance between symmetric and asymmetric cell divisions (Lechler and Fuchs, 2005; Taddei et al., 2008). Furthermore, ECM distribution affects migration of cancer cells and immune cells. During tumor progression, wavy collagen fibers become straightened and align perpendicular to the tumor boundary (Provenzano et al., 2006). It has been shown that linear collagen fibers oriented perpendicular to the tumors facilitate high-speed migration of breast cancer cells and paired macrophages to promote metastasis to distant organs (Roussos et al., 2011).

### Mechanical Properties

Tumor ECM is typically stiffer than normal tissue ECM due to overexpression of many ECM components (e.g., collagens I, II, III, V, IX, and XI, heparan sulfate proteoglycans) and ECM-modifying enzymes [e.g., lysyl oxidase (LOX)] (Levental et al., 2009). Mechanical properties conferred by ECM stiffness are transmitted to CSCs through the formation of focal adhesions and subsequent activation of mechanotransduction pathways (e.g., Rho/ROCK, YAP/TAZ). ECM stiffness plays a crucial role in regulating stem cell self-renewal and differentiation. Several studies have demonstrated that ECM stiffness directs human mesenchymal stem cells (MSCs) and neural stem cells to differentiate into different cell lineages (Engler et al., 2006; Saha et al., 2008; Winer et al., 2009). Human MSCs cultured on hydrogel with an elastic modulus very similar to bone marrow, exhibit enhanced self-renewal and multipotency (Winer et al., 2009). In the case of melanoma CSCs, three-dimensional (3D) soft fibrin matrices promote histone 3 lysine residue 9 (H3K9) demethylation and increase SOX2 expression and self-renewal, whereas stiff matrices exert the opposite effects (Liu et al., 2012; Tan et al., 2014). Conversely, breast CSCs increase CSC marker expression on stiff matrix through integrin linked kinase (ILK) signaling (Pang et al., 2016; You et al., 2016), suggesting that the effect of matrix stiffness on stemness is cancer type specific.

## ECM Modulates Biochemical Cues to Drive Cancer Stemness

### EMT/De-Differentiation

The ECM can provide external cues that induce EMT, one of the cellular transformation processes that has been shown to route some cancer cell types from a differentiated to a stem cell state (Mani et al., 2008). Collagen I has been shown to induce EMT through activation of ILK and subsequently NF-κB-dependent inactivation of GSK-3β (Medici and Nawshad, 2010), along with the nuclear translocation of β-catenin (Li et al., 2010). Collagen XVII and laminin-5 can also induce EMT-driven cancer stemness through the activation of FAK/Akt paired with inhibition of GSK-3β (Liu et al., 2018). The induction of EMT and CSC phenotypes by the ECM seems to be driven by a master regulator, Akt. Akt activation, which can be achieved via intracellular focal adhesion proteins such as FAK and ILK, subsequently modulates the activity of downstream effectors. For instance, Akt can activate NF-κB, which has been shown to upregulate the expression the stemness genes SOX2, NANOG and KLF4 in breast and prostate cancer cells (Liu et al., 2010; Moreira et al., 2015). Akt, as well as ILK, can also inactivate GSK-3β, which increases the nuclear translocation of β-catenin, a transcription factor that is associated with stemness and is also an activator of NOTCH and Wnt signaling (Vadlamudi et al., 2005; Fang et al., 2010). Therefore, ECM regulates the switch between CSC and non-CSC states by inducing EMT.

### Self-Renewal/Maintenance

The ECM also promotes CSC self-renewal. In this regard, collagen I has been shown to preserve stemness in malignant and non-malignant stem cells by activating transcriptional programs that induce self-renewal (Kirkland, 2009; Suh and Han, 2011). Binding of collagen to α2β1 integrin results in the nuclear translocation of Bmi1, a stemness-inducing transcription factor downstream of Hedgehog signaling. Studies have shown that Bmi1 is a transcriptional target of Gli1, a stemness related gene, and that FAK/Ras signaling enhances the expression of Gli1 (Goel et al., 2013). Akt/p-S6K1 signaling has also been shown to play a regulatory role in activity of Gli1 (Wang et al., 2012). Laminin and fibronectin signaling also plays a crucial role in CSC self-renewal. Laminin 511 can sustain breast cancer stemness through activation of α6β1 integrin, in a TAZ-dependent manner (Chang et al., 2015). TAZ expression and nuclear localization induce the expression of the stemness transcription factors, OCT4, SOX2 and NANOG in non-malignant and malignant cells (Varelas et al., 2008; Chang et al., 2015; Xiao et al., 2015). Fibronectin’s extra domain A (EDA) has also been demonstrated to positively regulate CSC self-renewal through activation of α9β1 integrin/FAK/ERK/Akt/β-catenin pathway (Ou et al., 2013).

### Growth Factor Reservoir and Release

The ECM might serve as a reservoir for factors that aid in the sustenance of CSCs. Embryonic stem cells (ESCs) have been shown to utilize matrix metalloproteases 1 (MMP1) to release ciliary neurotropic factor (CNTF) from an ESC-derived matrix, which enhances ESC self-renewal though JAK/STAT3 signaling (Przybyla et al., 2013), a pathway that has also been implicated in promoting self-renewal of breast CSCs (Wang et al., 2018). Hematopoietic stem cells (HSCs) also upregulate MMP-9 to release soluble kit-ligand, also known as stem cell factor (SCF), which promotes survival signaling and chemoresistance in many types of cancers (Foster et al., 2018). CSCs are thought to remodel their matrices more significantly than their non-cancer stem cell counterparts (Raja et al., 2015) as CSCs upregulate expression of different MMPs. This may enable them to effectively degrade and remodel ECM matrices (Inoue et al., 2010; Long et al., 2012) to release growth factors and cytokines to promote their survival.

### Metabolic Reprogramming and Autophagy

The ECM serves as a functional repository for a plethora of factors that dynamically modulate the tumor microenvironment to promote CSC metabolism. Focal adhesion formations transduce ECM signaling into the tumor cells and activate the PI3K pathway which increases glycolysis, in addition to activating glutamine signaling in a Ras- and Myc- dependent manner. Furthermore, a stiff ECM acts as a driver of glycolysis in CSCs (Pickup et al., 2014). On the contrary, accumulating evidence suggests that CSCs also utilize OXPHOS, fatty acid oxidation and glutaminolysis (Sancho et al., 2016; Martinez-Outschoorn et al., 2017). In this regard, it has been demonstrated that CSCs with high telomerase activity upregulate glycolysis and OXPHOS in lung and ovarian cancers (Bonuccelli et al., 2017). Given the diversity of tumors and their microenvironments, it is possible that based on the availability of nutrients, CSCs can manipulate their metabolism. For example, while CSCs in a hypoxic microenvironment may survive by means of glycolysis, CSCs in a normoxic environment use oxidative metabolism. Furthermore, CSCs utilize metabolites secreted by cancer-associated fibroblasts such as lactate and ketone bodies to fuel OXPHOS (Nazio et al., 2019). Recycling of nutrients via autophagy is another way by which CSCs not only self-renew but also acquire drug resistance (Mowers et al., 2018). Autophagy impairment downregulates the expression of CSC markers and consequently the CSC self-renewal capacity in breast, liver, ovarian and pancreatic cancers, osteosarcoma and gliobastoma (Nazio et al., 2019). ECM-receptor ligation has been shown to induce autophagy (Neill et al., 2014; Kawano et al., 2017). Collagen VI, a promoter of tumorigenesis (Chen et al., 2013) and a supporter of stem cell niches (Urciuolo et al., 2013), also functions as an autophagy inducer in skeletal muscle stem cells by functionally interacting with decorin, a small leucin-rich proteoglycans (SLRP) that has been shown to induce stemness in glioblastoma (Farace et al., 2015). A growing number of studies indicate that collagen VI directly maintains CSCs by activating the Akt–GSK-3β–β-catenin–TCF/LEF axis, which is required for activation of autophagy (Fan et al., 2018). Decorin signaling, independent of Collagen VI, can also maintain stemness of trophoblasts and prevent their differentiation (Nandi et al., 2018).

## Role of Hypoxia in ECM-Derived Cancer Stemness

Solid tumors frequently contain highly hypoxic regions and tumor hypoxia is positively associated with poor prognosis. Hypoxic tumor cells express stem cell markers, are highly undifferentiated and exhibit enhanced clonogenic potential *in vitro* and tumor initiating potential *in vivo* (Desplat et al., 2002; Jogi et al., 2002; Das et al., 2008; Kim et al., 2009). Furthermore, hypoxia can lead to increased ECM deposition and remodeling. Histological studies on clinical tumor samples have shown increased collagen deposition resulting in fibrosis in hypoxic regions of tumors (Shekhar et al., 2003). In addition to cancer cells, fibroblasts cultured under hypoxic conditions show increased type I procollagen α1 mRNA (Falanga et al., 1993; Tamamori et al., 1997; Norman et al., 2000). Abrogating HIF1α expression inhibits collagen deposition from both breast cancer cells and fibroblasts *in vitro* and *in vivo* (Gilkes et al., 2013a,b, 2014; Xiong et al., 2014). ECM remodeling enzymes such as LOX, LOX-like protein 2 (LOXL2), LOXL4, MMP2, MMP9 and MMP14 and growth factors inducing collagen deposition (e.g., VEGF) are HIF-regulated genes that are involved in tumor fibrosis (Gilkes et al., 2014). Since all these factors have been previously implicated cancer stemness, it is not surprising that the ECM acts a functional conduit for hypoxia-derived signals that foster cancer stemness.

## ECM Modulates Immune Surveillance in CSC Microenvironment

Extracellular matrix can profoundly influence recruitment of immune cells into the tumor microenvironment. CSCs can evade immune surveillance by altering this microenvironment to favor their survival. For example, ECM drives the activation of pro-survival pathways such as PI3K/AKT, which has been shown to facilitate immune evasion in CSCs (Dituri et al., 2011). ECM proteins can recruit immunosuppressive cells such as tumor-associated macrophages (TAMs) (Stahl et al., 2013; Lu et al., 2014) and regulatory T cells (Bollyky et al., 2011) that have been known to promote CSC survival, while simultaneously blocking the recruitment of antitumorigenic immune cells such as cytotoxic T cells (O’Connor et al., 2012). In addition, the ECM composition can dramatically modulate the activation state of the tumor infiltrating immune cells. For instance, a stiff collagen-rich or POSTN-rich ECM allows macrophage polarization to a pro-tumorigenic M2 phenotype (Wesley et al., 1998; Zhou W.C. et al., 2015). Following recruitment, the M2 macrophages activate several CSC survival signaling pathways including Src, NF-κB (Lu et al., 2014), STAT3/SOX2 (Yang et al., 2013) and Hedgehog (Jinushi et al., 2011). ECM can also impair proliferation and activation of T cells, that are required for capturing and killing CSCs (Di Tomaso et al., 2010). A collagen-rich ECM can inhibit T-cell proliferation and activation through type I collagen-dependent fusion of LAIR receptors (Meyaard, 2008; Frantz et al., 2010) in addition to sequestering growth factors required for T cell proliferation (Meyaard, 2008; O’Connor et al., 2012). Furthermore, TAMs (Martinez and Gordon, 2014) and neutrophils (Yakubenko et al., 2018) that can selectively reorganize the ECM to promote malignant growth of cancers are preferentially recruited to the microenvironment.

## CSC Targeting Therapies

Currently, there are several inhibitors targeting various aspects of ECM-induced cancer stemness that are undergoing clinical testing. For example, the CD47 blocking protein TTI-621 (Petrova et al., 2017) is currently being assessed in a number of phase I clinical trials (NCT03013218, NCT02663518, NCT02216409, NCT02678338) for various types of cancers. Other groups have targeted FAK with the inhibitor VS-6063 (Defactinib) (Lin et al., 2018), which has completed clinical phase I and II trials (NCT01778803, NCT01943292, NCT01951690) with one of those clinical trials assessing for CSCs as an endpoint (NCT01778803). Other inhibitors of stemness-related molecules further downstream of ECM signaling are also being tested in clinical trials, such as the STAT3 inhibitor BBI-608 (Sonbol et al., 2019) in a phase II trial that will test for presence of CSC as an endpoint (NCT02279719) and in a phase III clinical trial aimed at reducing CSCs by targeting phosphorylated Stat3 positive cancer cells (NCT02753127). The β-catenin pathway inhibitors PRI-724 and CWP232291 (Tai et al., 2015) are currently being tested in two phase I clinical trials (NCT01764477, NCT01398462). Inhibition of the Hedgehog pathway with the inhibitor GDC-0449 (Vismodegib) (Basset-Séguin et al., 2017), is also currently being clinically evaluated in a phase II trial which will test for the presence of pancreatic CSCs (NCT01088815).

## Challenges and Conclusion

Although the above drugs may effectively reduce the number of CSCs, there are still many potential challenges that ECM components in a tumor microenvironment may set that could interfere with an otherwise successful treatment regimen. Firstly, ECM proteins have been shown to act as a physical barrier, making drug delivery to cancer cells more difficult. Secondly, ECM proteins can de-differentiate non-CSCs into CSCs, which makes eliminating all CSCs more challenging. Thirdly, ECM plays a role in modulating immune cell recruitment, hence, potential immunotherapeutic strategies could be hindered by dysregulated ECM components. Finally, the ECM has a very complex and dynamic nature: different ECM molecules are expressed in a time and tissue-specific manner where various isoforms of the same molecule can play opposing functions in cancer stemness in a context-dependent manner. Considering these concerns, it is crucial that future studies further elucidate the role of ECM components on cancer stemness in order to design therapies that effectively eradicate all CSCs.

## Author Contributions

All authors conceptualized the content and wrote the manuscript.

## Conflict of Interest Statement

The authors declare that the research was conducted in the absence of any commercial or financial relationships that could be construed as a potential conflict of interest.
